# High-grade pancreatic intraepithelial neoplasia occurring in heterotopic pancreas of the gastric antrum: A case report

**DOI:** 10.1097/MD.0000000000045438

**Published:** 2025-10-24

**Authors:** Huikun Chen, Yanjun Chen, Wanqiong Shu, Guanghong Huang

**Affiliations:** a Affiliated Guangdong Hospital of Integrated Traditional Chinese and Western Medicine of Guangzhou University of Chinese Medicine, Foshan City, Guangdong Province, China.

**Keywords:** endoscopic submucosal dissection, endoscopic ultrasonography, heterotopic pancreas, high-grade pancreatic intraepithelial neoplasia, precancerous lesions

## Abstract

**Rationale:**

Heterotopic pancreas (HP) is a rare congenital developmental anomaly in which pancreatic tissue appears in a location other than the normal anatomical location of the pancreas. Most cases are no specific symptoms before surgery, and the pathological features are mostly benign, while malignant cases are rarely reported. HP with Pancreatic Intraepithelial Neoplasia (PanIN) is particularly rare in the literature. At present, the treatment of HP is controversial, especially when the lesion lacks obvious malignant characteristics, making it difficult to decide on surgical intervention.

**Patient concerns:**

A 47-year-old male was admitted to the hospital due to abdominal pain. He had been diagnosed with antral mass, cardiac mass, gastric retention, and chronic gastritis by gastroscopy in another hospital due to abdominal pain, which was managed nonsurgically. He denied any personal or family history of tumors.

**Diagnoses:**

After admission, the physical examination was normal, and the laboratory examination showed that CA72-4 and triglyceride were slightly elevated, and the rest were normal. Endoscopic ultrasonography revealed a submucosal protrusion on the posterior wall of the antrum, located in the muscularis mucosa and submucosa, approximately 10.7 × 7.7 mm in size, with relatively clear boundaries, and containing glandular duct-like structures. Color Doppler ultrasound did not show obvious color blood flow echoes within the lesion. The remaining mucosal layer and outer membrane layer were smooth.It was considered to be a HP, without obvious high-risk features.

**Interventions:**

Given the patient’s discomfort with abdominal pain, endoscopic submucosal dissection was performed, and a tissue sample was obtained for pathological testing.

**Outcomes:**

Pathological findings revealed HP tissue with moderate to severe atypia, consistent with high-grade PanIN (PanIN-3).

**Lessons:**

This case illustrates that PanIN-3 can occur even in small HP lesions without obvious malignant imaging features. Therefore, for patients with significant symptoms or uncertain diagnoses, endoscopic submucosal dissection may be considered as a personalized diagnostic and therapeutic intervention to effectively mitigate potential complications and the risk of progression to pancreatic ductal adenocarcinoma .

## 1. Introduction

Heterotopic pancreas (HP), also known as aberrant or accessory pancreas, is a congenital anomaly characterized by the growth of pancreatic tissue outside its normal location, with no anatomical or vascular connection to the main pancreas. HP has been reported in 0.55% to 13.7%, 0.25% and about 1.2% of autopsy materials, abdominal surgery and gastrectomy, respectively.^[1]^At present, there is no clear conclusion on the mechanism of HP, most scholars believe that it is caused by abnormal pancreatic embryonic development.

Due to its subepithelial location, it is difficult to obtain a suitable tissue specimen from HP via traditional superficial endoscopic biopsies. According to the guidelines of the European Society of Gastrointestinal Endoscopy, endoscopic ultrasonography (EUS) is recommended as the best tool for characterizing subepithelial lesions (SEL).^[2]^ If gastroscopy reveals the “central indentation sign”, the opening of the pancreatic duct into the stomach, this is a key imaging feature for diagnosing HP, although it is not observed in all cases.^[3]^ HP is usually asymptomatic and often discovered incidentally. However, it may become symptomatic when complicated by inflammation, hemorrhage, obstruction, or malignant transformation, depending on the anatomical location and size of the lesion.^[4,5]^

The malignant transformation rate of HP is 0.7% to 1.8%,^[6,7]^ with pancreatic ductal adenocarcinoma (PDAC) representing the predominant malignancy.^[8]^ The rising incidence of PDAC is projected to make it the second leading cause of cancer-related mortality. PanIN, defined as a spectrum of premalignant ductal epithelial lesions progressing from low-grade dysplasia (PanIN-1) to carcinoma in situ (PanIN-3), is the most common precursor to PDAC.^[9]^ While PanIN lesions are prevalent in the native pancreas, their occurrence in HP is rare, predominantly as low-grade (PanIN-1/2). High-grade PanIN (PanIN-3), corresponding to severe dysplasia or carcinoma in situ, is exceptionally rare in HP. Despite being noninvasive, histomorphological and molecular evidence suggests that PanIN-3 lesions carry the highest risk of progression to invasive carcinoma.^[10]^

Currently, there is no consensus on the management of HP. Therapeutic options primarily include surgical resection, endoscopic intervention, and conservative approaches. Some authors advocate surgical excision for symptomatic HP or lesions incidentally identified during procedures to prevent complications,^[11]^ while others propose regular surveillance for asymptomatic patients.^[12]^Incidentally detected HP lesions without high-risk features may be managed conservatively without resection or biopsy.^[13]^ This article presents a rare case of PanIN-3 arising in gastric antral HP, detailing its clinical manifestations, imaging characteristics, and management, thereby addressing the paucity of literature on high-grade dysplasia associated with HP.

## 2. Case report

The patient is a 47-year-old male with a history of hypertension and no relevant personal or family medical history. He previously visited the gastroenterology department of another hospital due to epigastric pain lasting over 4 months, where Esophagogastroduodenoscopy (EGD) revealed an antral protrusion (indeterminate nature); a SEL at the gastroesophageal junction (indeterminate nature); gastric retention; and chronic superficial gastritis. Symptoms improved with conservative management, and the patient was discharged.

Three months later, the patient experienced mild epigastric pain and discomfort again, without nausea, vomiting, melena, or abdominal distension, and denied weight loss. Physical examination was unremarkable. Laboratory tests showed elevated CA72-4 (6.23 U/mL; reference: 0–6 U/mL), normal levels of CA50, AFP, CEA, and CA19-9, and elevated triglyceride levels (2.89 mmol/L; reference: <1.7 mmol/L). Liver and kidney function, electrolytes, blood counts, coagulation, urinalysis, and stool studies were within normal limits.

**Figure 1.**
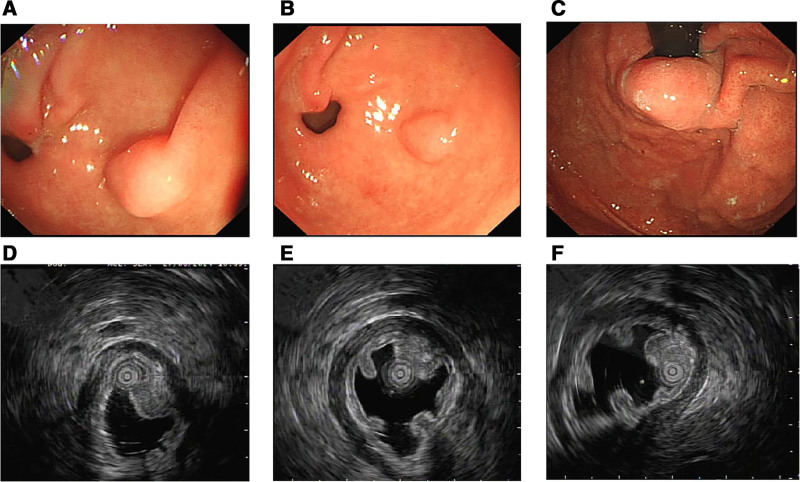
(A and B) show a submucosal protrusion in the gastric antrum under white light endoscopy, with a smooth mucosal surface and a slight depression in the center. (D–F) display the gastric antral protrusion under endoscopic ultrasound (EUS), which is located in the muscularis mucosa and submucosa, presenting as a slightly hyperechoic lesion with a size of approximately 10.7 mm × 7.7 mm. The lesion has relatively clear borders, while the demarcation from the muscularis propria is unclear in some layers. The remaining mucosal and outer layers are smooth. (F) reveals that the lesion contains duct-like structures. (C) shows a submucosal tumor at the cardiac orifice.

EUS identified a 10.7 × 7.7 mm submucosal lesion at the posterior gastric antrum with smooth overlying mucosa and central depression. The lesion exhibited a slightly hyperechoic area localized to the muscularis mucosa and submucosa, with partial blurring of the boundary with the muscularis propria, and contained glandular duct-like structures. Color Doppler imaging showed no intralesional vascularity. The mucosal and serosal layers were intact, consistent with HP (Fig. 1A, B, D–F). No high-risk features were observed.

**Figure 2.**
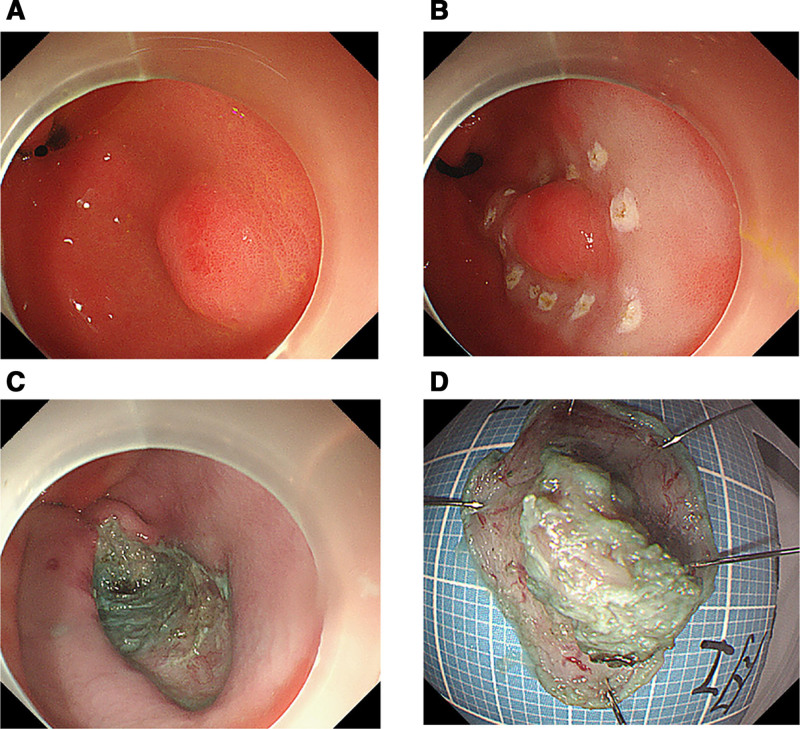
(A–D) Endoscopic submucosal dissection (ESD): The lesion was located in the gastric antrum, with a size of approximately 10.7 mm × 7.7 mm. The obtained specimen measured about 20 mm × 15 mm, and the lateral and deep margins were negative.

**Figure 3.**
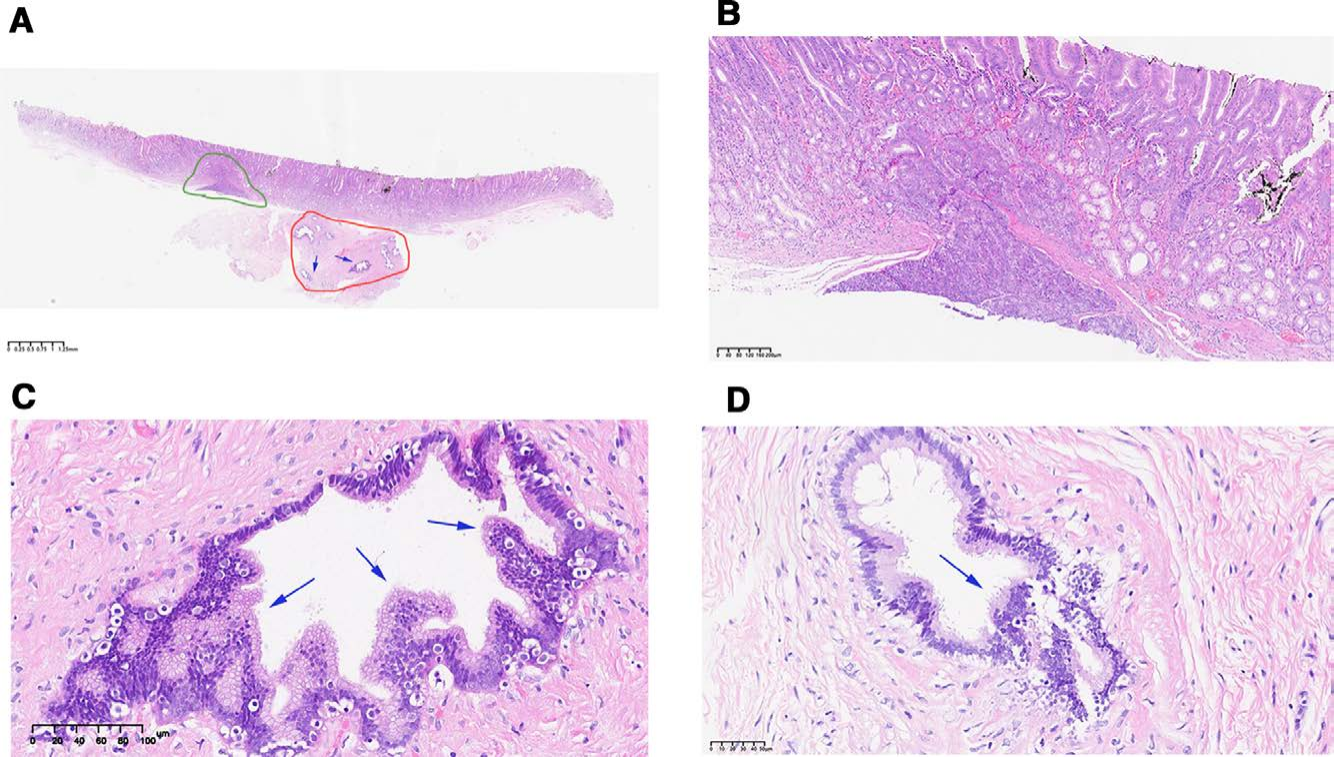
HE staining results: (A) is a panoramic view of the pathological section (scale bar = 1.25 mm). Green circle: Origin of heterotopic pancreas (HP); Red circle: Area of HP ductal epithelium with pancreatic intraepithelial neoplasia (PanIN); Blue arrow: Area with PanIN-3. (B) shows HP tissue located in the submucosa, extending upward to the deep mucosa; the mucous glands in the deep tissue are considered the origin of HP ducts (scale bar = 200 μm). (C and D) show HP ductal epithelium with cellular atypia and papillary structure formation (consistent with intraepithelial neoplasia, PanIN-3), which is confined to the ductal epithelium without evidence of stromal invasion (scale bar = 100 μm for [C]. scale bar = 50 μm for [D]).

Given the patient’s symptoms of abdominal pain, resection was performed using endoscopic submucosal dissection (ESD) (Fig. 2A–D). During the operation, the lesion was marked with an argon plasma coagulator. A submucosal injection (0.9% normal saline + methylene blue injection + epinephrine) was administered to elevate the lesion, followed by incision of the mucosal and submucosal layers using a disposable mucosal incision knife. Dissection was performed down to the muscularis propria, with assistance from traction using titanium clips and dental floss. Blood vessels were managed with a hot biopsy forceps (intraoperative bleeding was approximately 1 mL). After complete resection of the lesion, the wound surface was closed with titanium clips, and the total duration of the operation was 1 hour and 20 minutes. The resected specimen was routinely sent for pathological examination. Postoperatively, the patient was kept on fasting for 2 days and received intravenous treatment including rabeprazole (20 mg, qd) for acid suppression and oral teprenone (50 mg tid) for gastric mucosa protection. Mild pharyngeal discomfort occurred on the first postoperative day, which was relieved with Jinhoujian Spray. Abdominal pain resolved on the second postoperative day. The specimen resected by ESD measures 2.2 × 1.5 × 0.2 cm. A protuberance with a size of 1.1 × 0.6 × 0.5 cm is seen in the center of the mucosa, with a relatively clear boundary and a rough surface. The closest distance to the edge is 0.3 cm, the closest distance to the protuberance base is 0.3 cm, and the closest distance to the resection margin is 0.2 cm. The pathological results showed that the sections contained HP tissue (Fig. 3A and B).Some glands exhibited cellular atypia and formed papillary structures, which were considered to be intraepithelial neoplasia arising from the ductal epithelium. Most of the lesions were low-grade, with focal high-grade lesions (PanIN-3). Microscopically, the lesions were confined to the ductal epithelium, and the lesion margins were negative (Fig. 3C and D). No immunohistochemical or molecular tests were performed. The patient’s abdominal pain was relieved after symptomatic treatment, and the patient was followed up in the outpatient clinic. Four months later, the patient underwent submucosal tunneling endoscopic resection to remove the previously identified SEL at the gastroesophageal junction (Fig. 1C), measuring approximately 30 × 15 mm in size. Pathology suggested leiomyoma, and immunohistochemical results confirmed a benign lesion. Up to now, the patient has been followed up for 5 months, reporting no obvious discomfort, with no gastroscopy performed.

## 3. Discussion

Most HP are benign, usually asymptomatic, and are found incidentally in other examinations. HP can occur in any part of th e digestive tract. Most HP occur in the stomach (especially the antrum), duodenum, and proximal jejunum,^[14]^ and may also occur in Meckel’s diverticulum, ileum, spleen, lung, liver, gallbladder, esophagus, umbilical cord, and other parts.^[15,16]^ At present, studies on HP are mainly limited to case reports.

The characteristic EUS features of HP, including ill-defined margins, heterogeneous echogenicity, anechoic zones, fourth-layer thickening, and localization within the second, third, and/or fourth layers, are pivotal for preoperative diagnosis.^[17,18]^ In our case, considering the appearance of the mass and the EUS findings, we considered the possibility of HP. It has been reported that lesions > 1.5 cm in diameter have clinical significance, while HP tissue > 2 cm may cause chronic symptoms, with symptoms correlated to lesion size and mucosal involvement.^[19]^ Pain is one of the most common symptoms.^[20]^ In general, the mucosal appearance of SEL is normal. It has been reported that congestion, erosion, or ulceration of the mucosa overlying the tumor increases the risk of malignant transformation in SEL, and for SEL with intact overlying mucosa and a size < 2 cm, regular serial endoscopic follow-up may suffice.^[21]^ The patient was a middle-aged man with dull epigastric pain. EUS detected a 10.7 × 7.7 mm solid submucosal mass in the gastric antrum, with intact surface mucosa on endoscopy. One possible explanation for the pain is inflammation of the involved tissue.^[22]^ In our patient, the gastric antral mass had an intact mucosal appearance, and the lesion diameter was < 1.5 cm. The patient’s abdominal pain was mild, and no evidence of ulceration or inflammation was detected. Therefore, no complications or signs of malignant transformation were found, and it is not clear whether the pain was caused by HP.

The malignant features of SEL observed by EUS included echogenic lesions > 3mm, cystic space > 4 mm, irregular boundaries, and the presence of adjacent lymph nodes.^[23]^ The lesion in our case showed slightly hyperechoic signals on endoscopy, with indistinct boundaries with the muscularis propria in certain layers. It was located between the muscularis mucosa and submucosa, while the remaining mucosal and adventitial layers appeared smooth. Color Doppler ultrasound revealed no significant vascular signals within the lesion. The lobular structure of HP acinar tissue can make margins appear poorly defined, and although our patient did not have these malignant features, the decision was made to remove the lesion using ESD because of the patient’s symptoms of abdominal pain.

Of interest, we found PanIN-3 in the patient on histological examination. PanIN is the most important and well-known precancerous lesion of PDAC,^[24]^ and its morphological and genetic abnormalities highly overlap with PDAC.^[25]^ In addition to PanIN, intraductal papillary mucinous tumors and mucinous cystic tumors are also important precancerous lesions leading to the development of pancreatic cancer.^[26]^ Among them, PanIN is a microscopic lesion, while intraductal papillary mucinous tumors and mucinous cystic tumors are macroscopic.^[24]^ PanIN-3 is the most direct precancerous lesion of PDAC. If it can be found at this stage or earlier, timely intervention and treatment will significantly improve the possibility of cure. Unfortunately, because PanIN is difficult to detect using current technical means, it is essential to understand the biological behavior and molecular alterations of PanIN during the progression from PanIN to PDAC. At present, there have been a lot of studies on the orthotopic pancreas. However, it has been reported in the literature that there is a possibility that the intraepithelial neoplasia in HP and its similar lesions in the orthotopic pancreas do not follow the same pathway, or at least some of these lesions may follow a pathway similar to that of PDAC.^[25,27]^ The molecular mechanisms underlying the development of PanIN-3 in HP were not thoroughly investigated in this case. Currently, the number of reported cases of PanIN arising in HP remains limited, and existing evidence is insufficient to clarify its precise pathogenic mechanisms and progression patterns. Future multicenter studies with larger sample sizes are warranted to further validate these hypotheses.

At present, only 3 cases of HP with PanIN-3 without progression to adenocarcinoma were obtained through PubMed literature search (Table 1).^[28–30]^ The 2 cases that could be seen were both asymptomatic, and the diameter of the mass was >2 cm, and the covered mucosa was intact. Our case suggests that PanIN-3 may occur even in small-sized HP, indicating the need to pay attention to its malignant potential. In such cases, ESD can provide important references for clinical decision-making. The European Society of Gastrointestinal Endoscopy recommends that ESD be used as the preferred treatment for most superficial gastric lesions,^[2]^ and in particular, for SELs with a diameter of <20 mm where a definitive diagnosis cannot be achieved despite attempts, endoscopic resection may be chosen to avoid unnecessary long-term follow-up.^[31]^ The American College of Gastroenterology recommends that patients should enroll in some form of surveillance program when a tissue diagnosis and/or resection of the SEL has not been performed. However, there is insufficient evidence to provide clear recommendations regarding the surveillance interval when resection is not carried out.^[32]^ Several authors reported the application of ESD in the treatment of HP. Zhen et al conducted a retrospective study on 49 patients with gastric HP, evaluated the value of laparoscopic resection and ESD, and concluded that ESD in the treatment of gastric HP has become an important minimally invasive treatment technique, which is safe and feasible.^[33]^ Zhou reported the application of ESD in the treatment of 93 cases of HP, and the results of the study showed that ESD for gastric HP performed by an experienced endoscopist was a safe and feasible technique with good prognosis.^[34]^ No obvious complications were observed in our patient after ESD treatment, and no significant discomfort was reported during the subsequent follow-up. The main limitation of this study is that the follow-up time is only 5 months, and the first endoscopic surveillance has not yet been completed, which cannot fully assess the risk of lesion recurrence and the durability of the treatment effect. Although follow-up is currently limited, we have recommended EGD ± EUS at 6 to 12 months; if the results are normal, then consider reexamination every 2 to 3 years for 1 to 2 times. After that, we will customize the plan according to the individual situation to further observe the long-term prognosis.

**Table 1:** Summary of cases of panin-3 in heterotopic pancreas without cancer.

Author and year	Age/sex	HP location/size	Presentation	Intervention	Pathology (PanIN grade)	Margins	Follow-up
Moon-Soo Lee et al^[28]^, 2013	67/male	Fundus of stomach/2.3 × 2.0 cm	No symptoms	A laparoscopic gastric wedge resection	PanIN-3	Not reported	Not reported
Daisuke Niino et al^[29]^, 2014	-	Jejunum/-	-	-	PanIN-3	-	-
HL Yan et al^[30]^, 2022	48/female	Body of stomach/3 × 2.5 cm	No symptoms	Laparoscopic resection	PanIN-3	Not reported	Not reported
Our patient, 2025	47/male	Antrum of stomach/1.07 × 0.77cm	Abdominal pain	ESD	PanIN-3	Negative	Follow-up Duration: 5 mo ; Examination Items & Results: Clinical symptom assessment (endoscopic examination not yet performed)–no discomfort reported; Plan: It is recommended that EGD ± EUS be conducted 6–12 months postoperatively

This table summarizes all reported cases of HP with PanIN-3 without invasive carcinoma identified through a PubMed search up to [September 2025].

ESD = endoscopic submucosal dissection, HP = heterotopic pancreas, PanIN = pancreatic intraepithelial neoplasia.

Early detection of precancerous lesions is the key to improving prognosis. PanIN-3 in HP is extremely rare, and it is difficult to identify PanIN before surgery. The manifestations and characteristics of our case may provide a new perspective for the early detection and intervention of precancerous lesions in HP. At present, there is no consensus on the treatment of HP, and personalized strategies need to be developed according to the specific conditions of patients. Imaging examinations such as EUS and upper gastrointestinal endoscopy are helpful for initial evaluation, and accurate identification of HP is very important in diagnosis, but the final diagnosis is still pathological diagnosis. Diagnostic surgery should be considered as an important means to evaluate the benign or malignant nature of tumor tissues, which is the reason for surgical treatment. ESD can be used as an important option.

## 4. Conclusions

This case demonstrates that PanIN-3 can occur in a small gastric HP with benign features. Therefore, for such seemingly benign lesions, clinical attention should still be paid to their malignant potential. ESD is an important method for the diagnosis and treatment of HP with symptoms or an unknown diagnosis, especially in experienced medical centers.

## Author contributions

**Conceptualization:** Huikun Chen.

**Data curation:** Wanqiong Shu.

**Supervision:** Yanjun Chen.

**Writing – original draft:** Huikun Chen.

**Writing – review & editing:** Guanghong Huang.
